# Murine gammaherpesvirus 68 glycoprotein 150 does not contribute to latency amplification in vivo

**DOI:** 10.1186/1743-422X-9-107

**Published:** 2012-06-09

**Authors:** Romana Ruiss, Shinji Ohno, Beatrix Steer, Reinhard Zeidler, Heiko Adler

**Affiliations:** 1 Research Unit Gene Vectors, Helmholtz Zentrum München-German Research Center for Environmental Health, Munich, Germany; 2 Institute of Molecular Immunology, Helmholtz Zentrum München-German Research Center for Environmental Health, Munich, Germany; 3 Department of Otorhinolaryngology, Klinikum der Universität München, Munich, Germany; 4 Present address: Department of Virology, Faculty of Medicine, Kyushu University, Fukuoka, 812-8582, Japan

**Keywords:** MHV-68, gp150, Latency amplification, Vaccination, Exosomes

## Abstract

**Background:**

Murine gammaherpesvirus 68 (MHV-68) is used as a model to study the function of gammaherpesvirus glycoproteins. gp150 of MHV-68, encoded by open reading frame M7, is a positional homolog of gp350/220 of EBV and of gp35/37 of KSHV. Since it had been proposed that gp350/220 of EBV might be a suitable vaccine antigen to protect from EBV-associated diseases, gp150 has been applied as a model vaccine in the MHV-68 system. When analyzing the function of gp150, previous studies yielded conflicting results on the role of gp150 in latency amplification, and disparities between the mutant viruses which had been analyzed were blamed for the observed differences.

**Results:**

To further develop MHV-68 as model to study the function of gammaherpesvirus glycoproteins in vivo, it is important to know whether gp150 contributes to latency amplification or not. Thus, we re-evaluated this question by testing a number of gp150 mutants side by side. Our results suggest that gp150 is dispensable for latency amplification. Furthermore, we investigated the effect of vaccination with gp150 using gp150-containing exosomes. Vaccination with gp150 induced a strong humoral and cellular immune response, yet it did not affect a subsequent MHV-68 challenge infection.

**Conclusions:**

In this study, we found no evidence for a role of gp150 in latency amplification. The previously observed contradictory results on the role of gp150 in latency amplification were not related to differences between the mutant viruses which had been used.

## Background

Infection of cells by herpesviruses is orchestrated by several viral glycoproteins which function either separately or within multiprotein-complexes to mediate virus entry [1]. Murine gammaherpesvirus 68 (MHV-68) has been extensively used as a model to study the function of gammaherpesvirus glycoproteins [2-5]. Glycoprotein 150 (gp150) of MHV-68 is encoded by open reading frame M7 [6] and has been shown to be virion-associated [7]. It is a positional homolog of gp350/220 of EBV and of gp35/37 of KSHV [6], and it has been shown to be important for virion release [8,9] and antibody evasion [10]. When studying the function of gp150, both de Lima et al. [9] and Stewart et al. [8] found that mutation of gp150 did not considerably affect lytic replication in vivo and in vitro, except from a moderate deficit in release of extracellular virus. However, Stewart et al. observed a reduction in splenomegaly and in the number of latently infected cells during latency amplification. In contrast, de Lima et al. reported normal latency amplification by gp150-deficient MHV-68. Clearly, to further develop MHV-68 as model for studying the function of gammaherpesvirus glycoproteins in vivo, it is important to know whether gp150 reliably contributes to latency amplification. Here, we present data supporting that gp150 is dispensable for latency amplification but it remains possible that it might play a role under different, so far ill-defined circumstances.

## Results and discussion

Previously, conflicting results have been published on the role of gp150 in latency amplification [8,9], and several hypotheses were raised to explain these discrepancies: i) Stewart et al. introduced a large deletion in the MHV-68 genome to disrupt gp150 which might have inadvertently disrupted additional viral functions, e.g. unknown latency transcripts [2]. On the other hand, the mutations made by de Lima et al. might allow the expression of a truncated but still functional gp150 protein [8]. ii) Stewart et al. investigated only a single mutant while de Lima et al. constructed two independent mutants. The latter can be considered to be a superior strategy to study a gene function [2]. Although Stewart et al. carefully analyzed their mutant by various standard procedures, and even analyzed the viral transcriptome by DNA oligonucleotide microarray, it is formally possible that subtle defects escaped their analysis. iii) Stewart et al. also constructed a BAC-based revertant but BAC-based revertants do not control for problems after virus reconstitution [2].

### gp150 and latency amplification

To address the above mentioned points, we re-evaluated whether gp150 plays a role during latency amplification. For this purpose, we analyzed the following viruses: i) the original mutant (vgp150Δ) which was constructed in our own laboratory and which has been described in Stewart et al. [8]; ii) a second gp150 mutant (vgp150Δ_2) which we independently reconstituted from the same BAC clone from which the original mutant vgp150Δ had been derived - to test for aberrations which might have occurred during reconstitution; iii) a M7^-^FRT mutant constructed exactly as described by de Lima et al. [9], to test whether the different size and position of the deletion affect the phenotype. All mutant viruses were compared to the revertant virus vgp150R, which had been constructed in our own laboratory and shown to behave exactly like parental (wild-type) virus [8].

**Figure 1:**
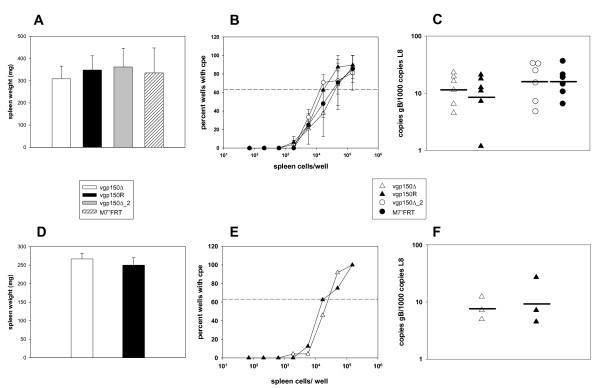
**gp150 does not contribute to latency amplification.A**) and **D**) Spleen weights, **B**) and **E**) *Ex vivo* reactivation of splenocytes and **C**) and **F**) Viral genomic load in the spleen. C57BL/6 mice (**A**-**C**) or Balb/c mice (**D**-**F**) were infected i.n. with 5×10^4^ PFU. At day 17 after infection, spleens were harvested and the spleen weights were taken (the spleen weight of uninfected mice of the same age was in the range of 80 to 120 mg; data not shown). Single splenocyte suspensions were prepared and analyzed in the ex vivo reactivation assay or used for DNA isolation for real time PCR analysis. Data shown in panels **A**) and **D**) are means + SD, in **A**) from 6 mice compiled from two independent experiments and in **D**) from three mice from a single experiment. Data shown in panel B) are means + SEM, derived from two experiments. Data shown in panel **E**) are derived from a single experiment. In each experiment, splenocytes from 3 mice per group were pooled. The dashed line in panels **B**) and **E**) indicates the point of 63.2% Poisson distribution, determined by nonlinear regression, which is used to calculate the frequency of cells reactivating lytic replication. In panels **C**) and **F**), each symbol represents an individual mouse and the bars represent the mean. The data in **C**) are compiled from 2 independent experiments, the data in **F**) are from a single experiment.

To analyze the role of gp150 on latency amplification, we infected C57BL/6 mice intranasally (i.n.) with 5 × 10^4^ PFU. At day 17 after infection, a time point when latency amplification is going on, spleens were harvested and the spleen weights (as a measure of splenomegaly) were determined. Furthermore, the numbers of latently infected cells were determined by a limiting dilution reactivation assay and the latent viral load was measured by quantitative real time PCR. As shown in Figure 1A-C, we did not observe significant differences in any of these parameters (two-tailed Student’s *t*-test). To rule out that this finding was specific for C57BL/6 mice, we additionally performed an experiment in BALB/c mice, comparing mutant virus vgp150Δ with its revertant (Figure 1D-F). Again, no significant differences were observed (two-tailed Student’s *t*-test). Thus, our data suggest that gp150 is not important for latency amplification and clearly show that the different results obtained in the previous studies by Stewart et al. [8] and de Lima et al. [9] are not caused by differences between the mutant viruses which had been used. This finding clearly differs from studies analyzing the role of the MHV-68 open reading frame M3. M3 is a broad-spectrum chemokine binding protein which inhibits chemokine action [11,12]. Disruption of M3 by insertion of a β-galactosidase expression cassette significantly compromised latency establishment [13], while a M3 mutant generated by insertion of a translational stop codon followed by a frame shift showed normal latency establishment [14]. In this case, the different observations have been attributed to the insertion of the β-galactosidase expression cassette which may have caused additional adverse effects not related to mutation of M3 [15]. Since the diverse results obtained for gp150 deletions are obviously not related to differences between the mutant viruses which had been used, we speculate that they might be due to differences of the host, the host environment or to subtle differences in the methods applied and time points analyzed. For example, we infected mice at 6 to 8 weeks of age with 5 × 10^4^ PFU, whereas Stewart et al. infected mice at 4 to 6 weeks of age with 4 × 10^5^ PFU [8]. While we analyzed splenomegaly by measuring the spleen weight and the number of ex vivo reactivating cells by ex vivo limiting dilution reactivation assay at day 17 after infection, Stewart et al. used the spleen cell number as a measure of splenomegaly and infectious center assays to determine the number of ex vivo reactivating cells at days 10, 14 and 21 after infection [8]. In addition, different experimental outcomes may correlate with specific properties of the respective mouse facilities. For example, moving Atg16L1-deficient mice from conventional barrier living conditions to an enhanced barrier facility has recently been shown to dramatically change their phenotype [16]. It was shown that this was due to the presence of a particular strain of norovirus in one of the facilities.

### gp150 and vaccination

**Figure 2:**
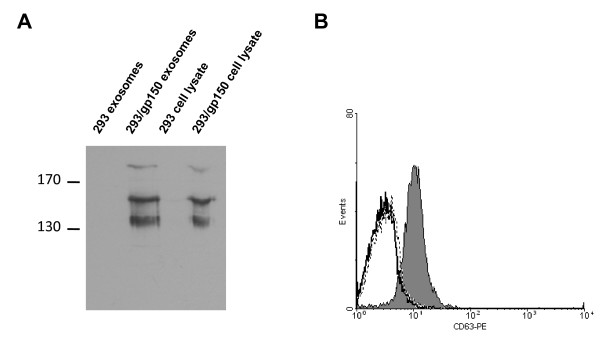
**Characterization of exosomes.** Exosomes were analyzed for overall gp150 content by Western Blot (panel **A**) and for gp150 surface content by FACS analysis (panel **B**). **A**) Gp150 was detected in purified exosomes and total cell lysates with polyclonal rabbit anti-MHV-68 antibody. Marker sizes (in kD) are indicated on the left. B) Gp150 on the surface of exosomes was detected by binding of exosomes to flow cytometry Protein G antibody binding beads coated with polyclonal rabbit anti-MHV-68 antibody. Binding of exosomes was visualized by staining for the exosome marker CD63 using a PE-conjugated anti CD63 monoclonal antibody. Solid black line: beads only; dotted black line: beads + 293 exosomes; grey histogram: beads + 293/gp150 exosomes.

**Figure 3:**
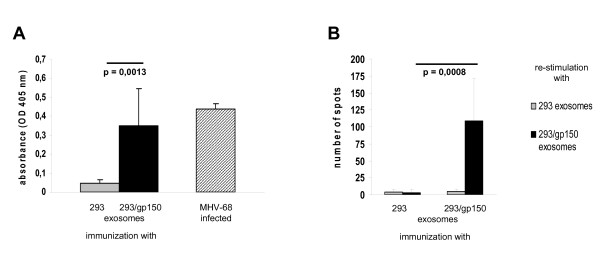
**Immune response after vaccination with gp150-containing exosomes.** Mice were vaccinated twice (day 0 and 14) with gp150-containing exosomes (293/gp150) or as a control, with exosomes prepared from untransfected 293 cells (293). Nineteen days after the second vaccination (day 33), sera were analyzed by ELISA for the presence of anti-gp150 antibodies (panel **A**). For comparison, antibody levels of MHV-68 infected mice (> 2 weeks after infection) are shown. The presence of gp150-specific T-cells was detected by re-stimulation of splenocytes with exosomes (+/− gp150) and subsequent quantification of activated T-cells by IFN-γ-ELISPOT (panel **B**). Data shown are means + SD derived from eight (293 exosomes), seven (293/gp150 exosomes) and two (MHV-68 infected) mice, respectively. The Student’s *t*-test (unpaired) was used for statistical analysis

**Figure 4:**
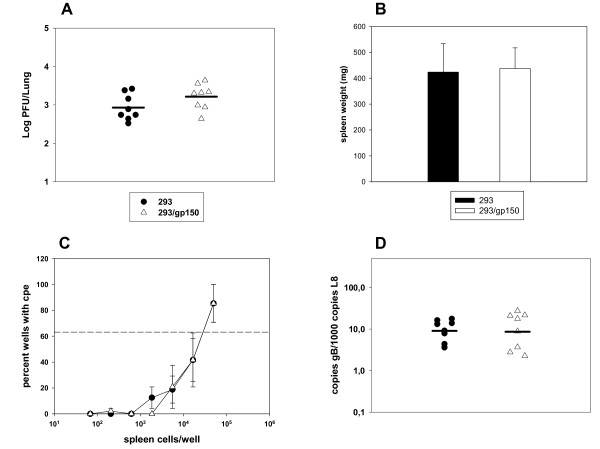
**Vaccination with gp150-containing exosomes does not affect a subsequent MHV-68 challenge infection.A**) Virus titers in the lung, **B**) Spleen weights, **C**) *Ex vivo* reactivation of splenocytes and **D**) Viral genomic load in the spleen. Mice were vaccinated twice (day 0 and 14) with gp150-containing exosomes (293/gp150) or as a control, with exosomes prepared from untransfected 293 cells (293). On day 28, mice were challenged by i.n. infection with 5 × 10^4^ PFU. **A**) Lytic replication was analyzed five days after infection (day 33) by determining virus titers in lung homogenates by standard plaque assay. Each symbol represents an individual mouse and the bars represent the mean. The data are compiled from 2 independent experiments. **B**) to **D**) Seventeen days after infection (day 45), spleens were harvested and the spleen weights were taken (panel **B**). Single splenocyte suspensions were prepared and analyzed in the ex vivo reactivation assay (panel **C**) or used for DNA isolation for real time PCR analysis (panel **D**). Data shown in panel B) are means + SD from 8 mice compiled from two independent experiments. Data shown in panel **C** are means + SEM compiled from two independent experiments. In each experiment, splenocytes from 5 and 3 mice per group, respectively, were pooled. The dashed line in panel **C**) indicates the point of 63.2% Poisson distribution, determined by nonlinear regression, which is used to calculate the frequency of cells reactivating lytic replication. In panel **D**), each symbol represents an individual mouse and the bars represent the mean. The data are compiled from 2 independent experiments. There were no significant differences in virus titers in the lung (panel **A**), spleen weights (panel **B**), *ex vivo* reactivation of splenocytes (panel **C**) and viral genomic load in the spleen (panel **D**). The two-tailed Student’s *t*-test was used for statistical analysis.

The MHV-68 model might be very useful to define effective strategies for gammaherpesvirus vaccination [17]. Since it had been proposed that gp350/220 might be a suitable vaccine antigen to protect from EBV-associated diseases [18], the positional homolog of MHV-68, gp150, has been used as a model vaccine in the MHV-68 system. If gp150 contributes to the extent of splenomegaly and to the frequency of latently infected cells during latency amplification in the spleen, as proposed by Stewart et al. [8], it would be reasonable to expect a reduction of both parameters after vaccination with gp150. Indeed, vaccination with a recombinant vaccinia virus expressing gp150 reduced both splenomegaly and the number of latently infected cells in the spleen during latency amplification while it did not reduce lung infection after i.n. MHV-68 challenge infection [8,19]. In a separate study, however, an approximately 10-fold reduction in the lytic virus load in the lungs but no reduction of splenomegaly and of the number of latently infected spleen cells after vaccination with dendritic cells pulsed with a MHC class II-restricted gp150 peptide was observed [20]. Since we did not observe a reduction in splenomegaly and in the number of latently infected cells during latency amplification in the spleen after infection of mice with MHV-68 lacking gp150, we re-evaluated the effect of vaccination with gp150. Specifically, we asked i) whether vaccination of mice with gp150 might induce an anti-gp150 immune response and if so, ii) whether this immune response influences a subsequent MHV-68 challenge infection. For vaccination, we used gp150-containing exosomes. Exosomes are small membrane vesicles which are released into the extracellular compartment during fusion of multivesicular bodies with the plasma membrane and are secreted by various cell types [21]. Exosomes expressing tumor antigens have been shown to be immunogenic, demonstrating the potential of exosomes as vaccines to generate antitumor responses [21,22]. We produced gp150-containing exosomes (293/gp150 exosomes) by transfection of HEK293 cells with an expression plasmid coding for gp150. Exosomes prepared from untransfected HEK293 cells (293 exosomes) served as control. While gp150 was readily detectable by Western Blot in 293/gp150 exosomes, it was not present in control 293 exosomes (Figure 2A). Consistent with data from Stewart et al. [19], we also detected the gp150 precursor gp130 and alternatively-glycosylated forms of higher molecular weight (Figure 2A). As shown by flow cytometry, 293/gp150 exosomes bound to beads coated with polyclonal anti-MHV-68 antiserum, demonstrating that gp150 is exposed on the surface of 293/gp150 exosomes (Figure 2B). To test the effect of vaccination with gp150, mice were vaccinated twice (day 0 and 14) with 10 μg 293/gp150 exosomes or, as a control, with the same amount of 293 exosomes. Nineteen days after the second vaccination (day 33), sera of immunized mice were analyzed by ELISA for the presence of anti-gp150 antibodies, and gp150-specific T cells were quantified by ELISPOT. Vaccination with 293/gp150 exosomes, but not with control 293 exosomes, induced both humoral (Figure 3A) and cellular gp150-specific immune responses (Figure 3B). The level of the humoral immune response induced by the 293/gp150 exosome vaccination was comparable to that after infection with MHV-68 (Figure 3A). Next, we challenged vaccinated mice by i.n. infection with 5 × 10^4^ PFU of MHV-68 on day 28 and analyzed both lytic and latent infection. Lytic replication was analyzed five days after infection (day 33) by determining lytic virus titers in lung homogenates by standard plaque assay. As shown in Figure 4A, immunization did not influence the amount of lytic virus present in the lungs of infected mice. This result is consistent with previous findings after immunization with a recombinant vaccinia virus expressing gp150 [8,19] but different to findings after vaccination with dendritic cells pulsed with a MHC class II-restricted gp150 peptide [20]. We also analyzed splenomegaly and the number of latently infected cells in the spleen seventeen days after infection (day 45). We found no evidence for an influence of vaccination with gp150 on splenomegaly (Figure 4B), on the number of reactivating cells as determined by limiting dilution reactivation assay (Figure 4C) and on the latent viral load as measured by quantitative real time PCR (Figure 4D). While these results are consistent with the data from Woodland et al. [20], they deviate from findings reported by Stewart et al. [19]. The latter might be due to differences of several parameters between the study by Stewart and our study: i) different immunization routes and schedules (subcutaneous vaccination with boost after 28 days vs. intraperitoneal (i.p.) vaccination with boost after 14 days); ii) age and strain of mice (4 week old BALB/c mice vs. 6–8 week old C57BL/6 mice); iii) time and dose of challenge infection (challenge with 4 × 10^5^ PFU 28 days after second vaccination vs. challenge with 5 × 10^4^ PFU 14 days after second vaccination) and iv) methods and time points used for read-out (virus neutralisation assay, spleen cell number and infectious center assay at days 10, 14, 21 and 28 after challenge infection vs. ELISA, ELISPOT, spleen weight and ex vivo limiting dilution reactivation assay at day 17 after challenge infection). Most importantly, while we used gp150-containing exosomes for vaccination, Stewart et al. used recombinant vaccinia virus expressing gp150. Recombinant vaccinia viruses expressing foreign antigens are powerful vaccines [23]. Yet, vaccination with gp150-containing exosomes also induced a considerable humoral and cellular immune response but did not influence a subsequent MHV-68 challenge infection. This is consistent with more recent findings by Gillet et al. [10], proposing that gp150 is not a significant neutralization target but rather acts as an immunogenic decoy.

## Conclusions

Taken together, our data suggest that gp150 is dispensable for latency amplification. Vaccination with gp150-containing exosomes did not affect a subsequent MHV-68 challenge infection, even though it induced a strong humoral and cellular immune response.

## Methods

### Cell lines and virus stocks

BHK-21 cells (ATCC CCL-10) were grown in Glasgow-MEM (PAN Biotech, Aidenbach, Germany) supplemented with 5% fetal calf serum (FCS), 5% tryptose phosphate broth, 2 mM L-glutamine, 100 U/mL penicillin and 100 μg/mL streptomycin. NIH3T3 cells (ATCC CRL-1658) and HEK293 cells (ATCC CRL-1573) were grown in DMEM (Invitrogen, Darmstadt, Germany) supplemented with 10% FCS, 2 mM L-glutamine, 100 U/mL penicillin and 100 μg/mL streptomycin. Working stocks of viruses were prepared as previously described [24]. Briefly, stocks were grown by infecting BHK-21 cells. After showing complete cytopathic effect (CPE), BHK-21 cells were harvested and the supernatant was used as working stock after two times freezing/thawing the cells and removing cell debris by centrifugation. Virus titers were determined by plaque assays. Briefly, 10-fold dilutions were incubated on BHK-21 cells for 90 min at 37 °C. After removing the inoculum, cells were incubated for 5 days at 37 °C with fresh medium containing methylcellulose. Cells were stained with 0.1% crystal violet solution to determine the number of plaques.

### In vivo experiments

Six to eight weeks old C57BL/6 or BALB/c mice were purchased from Charles River Laboratories (Sulzfeld, Germany) and housed in individually ventilated cages (IVC) during the MHV-68 infection period. Mice were infected intranasally (i.n.) with 5 × 10^4^ plaque forming units (PFU) of MHV-68. Prior to i.n. infection, mice were anesthetized with ketamine and xylazine. To determine lytic virus titers, the lungs were harvested and homogenized using the FASTPREP®-24 instrument (MP Biomedicals, Heidelberg, Germany). After two times freezing and thawing the homogenates, plaque assays were performed with 10-fold dilutions as described above. To determine the frequency of cells carrying virus reactivating from latency, spleens were harvested, single splenocyte suspensions were prepared and analyzed in an ex vivo limiting dilution reactivation assay as described [25]. Briefly**,** serial threefold dilutions of infected mouse splenocytes were plated on monolayers of 1 × 10^4^ low-passage NIH3T3 cells per well in 96-well tissue culture plates. 24 wells were plated per dilution (starting with 1,5 × 10^5^ cells/well). NIH3T3 cells were screened microscopically for a viral cytopathic effect (cpe) for up to 3 weeks. To differentiate between latently infected cells and infectious virus in the samples, serial threefold dilutions of spleen cells were plated before or after mechanical disruption of viable cells (by two freeze-thaw cycles). No infectious virus was detected in samples of mechanically disrupted cells (data not shown). Frequencies of reactivating cells were calculated on the basis of the Poisson distribution by determining the cell number at which 63.2% of the wells scored positive for CPE. All animal experiments were in compliance with protocols approved by the local Animal Care and Use Committee (District Government of Upper Bavaria; permit number 124/08).

### Measurement of latent viral load by quantitative real time PCR

Viral load in the spleens of infected mice was determined by quantitative real-time PCR using the ABI 7300 Real Time PCR System (Applied Biosystems, Foster City, CA) as described [26]. Briefly, DNA was extracted from spleen cells using the QIAmp DNA Mini Kit (Qiagen, Hilden, Germany) and quantified by UV spectrophotometry. Amplification of 100 ng of DNA per reaction was performed with Taqman universal PCR master mix and universal cycling conditions (Applied Biosystems, Foster City, CA). Using primers and probes as described [27], a 70 bp region of the MHV-68 glycoprotein B (gB) gene was amplified and viral DNA copy numbers were quantified. A standard curve was created using known amounts of a plasmid containing the *Hin*dIII-N fragment of MHV-68 encompassing the gB gene. The murine ribosomal protein L8 (rpl8) was amplified in parallel and used to normalize for input DNA between samples. The primer and probe sequences for L8 were as follows: Forward: 5’-CATCCCTTTGGAGGTGGTA-3’; Reverse: 5’-CATCTCTTCGGATGGTGGA-3’ and Probe: 5’-ACCACCAGCACATTGGCAAACC-3’. A standard curve for rpl8 was generated by serial 10 fold dilution of a plasmid containing rpl8 (RZPD clone IRAVp968B01123D6, RZPD, Berlin, Germany). The data are presented as viral genome copy numbers relative to the copy number of L8. The quantification limit was set at 50 copies per sample, according to published recommendations [28].

### Production and characterization of gp150-carrying exosomes

To generate gp150-carrying exosomes, 293 cells were transfected with an expression plasmid coding for gp150, and three days later, exosomes were prepared from the supernatant. Control exosomes were prepared in the same way from untransfected 293 cells. Briefly, exosomes were subjected to sequential centrifugation steps (300 g for 10 min, 5000 g for 10 min and 100.000 g for 120 min). The pelleted exosomes were washed and resuspended in PBS containing proteinase inhibitors (Complete Mini Proteinase Inhibitor Cocktail, Roche, Penzberg, Germany). The protein content was analyzed in a BCA protein microassay (Pierce, Rockford, USA). Exosomes were analyzed for overall gp150 content by Western Blot and for gp150 surface content by FACS analysis. For Western Blotting, 30 μg of exosomes were lysed in sample buffer, separated by SDS-PAGE and transferred onto a Hybond-ECL membrane (GE Healthcare, Munich, Germany). gp150 was detected by incubating the membrane with polyclonal rabbit anti-MHV-68 antibody [29], followed by peroxidase-conjugated donkey anti-rabbit antibody (Jackson Laboratories, Newmarket, UK). Blots were developed using ECL reagent (GE Healthcare, Munich, Germany) according to the instructions of the manufacturer. For FACS analysis, flow cytometry Protein G antibody binding beads (Polysciences Europe GmbH, Eppelheim, Germany) were coated with polyclonal rabbit anti-MHV-68 antibody for 30 min at room temperature. After washing with PBS and blocking with RPMI/10% FCS, beads were incubated with exosomes for two hours at room temperature. After washing, bound exosomes were detected by incubation with PE-conjugated anti CD63 monoclonal antibody (Immunotools, Friesoythe, Germany). CD63, a member of the tetraspanin family, is a marker protein for exosomes [21].

### Vaccination with exosomes and challenge infection

Mice were vaccinated twice (day 0 and 14) with gp150-containing exosomes (293/gp150 exosomes) or as a control, with exosomes prepared from untransfected 293 cells (293 exosomes). Vaccination was performed i.p. with a mixture of 10 μg of exosomes and 10 nmol CpG-ODN 1668 (InvivoGen, San Diego, USA) in a total volume of 200 μl PBS per mouse. Fourteen days after the second vaccination (day 28), mice were challenged with 5 × 10^4^ PFU of MHV-68.

### ELISPOT assay for determination of gp150-specific T cells

To determine gp150-specific T-cells, splenocytes from vaccinated mice were plated onto precoated ELISPOT plates in a densitiy of 10^6^ cells/well and restimulated in vitro with 10 μg of exosomes (+/− gp150). After 24 h, activated T-cells were quantified using a mouse IFN-γ-ELISPOT Kit (eBioscience, Frankfurt, Germany) as recommended by the manufacturer, and spots were counted by inspection under a dissecting microscope.

### ELISA for determination of gp150-specific antibodies

To determine gp150-specific antibodies in sera of vaccinated mice, Nunc Maxisorp plates (Nunc, Wiesbaden, Germany) were coated overnight with 3 μg/ml of MHV-68 lysate. The lysate was prepared by disruption of MHV-68 via dilution in PBS with 0.05% Triton X-100 as described [30]. After washing with PBS/Tween, plates were blocked with RPMI containing 10% FCS for 1 hour and washed again. Then, fifty-fold serum dilutions were incubated for two hours. After washing, bound antibody was detected with HRP-conjugated rat anti-mouse antibody (Promega, Mannheim, Germany) using TMB (Becton Dickinson, Heidelberg, Germany) as substrate and, after stopping with 1 M phosphoric acid, reading the absorbancy at 450 nm.

### Statistical methods

If not otherwise indicated, data were analyzed by two-tailed Student’s *t*-test.

## Competing interests

The authors declare that they have no competing interests.

## Authors’ contributions

RZ and HA conceived and designed the experiments. RR, SO, BS and HA performed the experiments. RR, SO, RZ and HA analyzed the data. HA wrote the paper. All authors read and approved the final manuscript.
